# Comparative proteomic analyses of Tartary buckwheat (*Fagopyrum tataricum*) seeds at three stages of development

**DOI:** 10.1007/s10142-022-00912-1

**Published:** 2022-11-11

**Authors:** Jiao Deng, Jiali Zhao, Juan Huang, Rebecca Njeri Damaris, Hongyou Li, Taoxiong Shi, Liwei Zhu, Fang Cai, Xiaona Zhang, Qingfu Chen

**Affiliations:** 1 Research Center of Buckwheat Industry Technology, Guizhou Normal University, Guiyang, 550001 China; 2 College of Life Science, Sichuan Agricultural University, Ya’an, 625014 China; 3 Department of Biological Sciences, Pwani University, Kilifi, 195-8010 Kenya

**Keywords:** Tartary buckwheat, Starch, Seed storage protein, Flavonoid, Regulation mechanism

## Abstract

Tartary buckwheat is among the valuable crops, utilized as both food and Chinese herbal medicine. To uncover the accumulation dynamics of the main nutrients and their regulatory mechanism of Tartary buckwheat seeds, microscopic observations and nutrient analysis were conducted which suggested that starch, proteins as well as flavonoid gradually accumulated among seed development. Comparative proteomic analysis of rice Tartary buckwheat at three different developmental stages was performed. A total of 78 protein spots showed differential expression with 74 of them being successfully identified by MALDI-TOF/TOF MS. Among them, granule bound starch synthase (GBSS1) might be the critical enzyme that determines starch biosynthesis, while 11 S seed storage protein and vicilin seemed to be the main globulin and affect seed storage protein accumulation in Tartary buckwheat seeds. Two enzymes, flavanone 3-hydroxylase (F3H) and anthocyanidin reductase (ANR), involved in the flavonoid biosynthesis pathway were identified. Further analysis on the expression profiles of flavonoid biosynthetic genes revealed that F3H might be the key enzyme that promote flavonoid accumulation. This study provides insights into the mechanism of nutrition accumulation at the protein level in Tartary buckwheat seeds and may facilitate in the breeding and enhancement of Tartary buckwheat germplasm.

**Supplementary Information:**

The online version contains supplementary material available at 10.1007/s10142-022-00912-1.

## Introduction

Buckwheat, belonging to the Polygonaceae family and genus *Fagopyrum*, has 21 species, among which, common buckwheat (*F. esculentum* Moench) and Tartary buckwheat (*F. tataricum* (L.)) are the main cultivated species in many parts of Asia, Europe, and America (Huda et al. 2021). Although buckwheat is a kind of pseudocereal crop plant, it has high nutrition value, and like other common crops, it contains starch, proteins, fats, minerals, vitamins, dietary fiber, among others (Bonafaccia et al. 2003; Joshi et al. 2020), and can be consumed as noodles, pancakes, cookies, sachima, and muffins in many countries (Sytar et al. 2016). Besides, buckwheat proteins consist of a balanced and reasonable composition of amino acids, including 8 amino acids that are necessary for humans. (Qin et al. 2010). Additionally, Tartary buckwheat contains high content of flavonoid metabolites in many tissues (Li et al. 2019b; Uddin et al. 2013; Li et al. 2022; Deng et al. 2021), which possess diverse health-promoting effects, such as anti-oxidative, anti-cancer, anti-tumor, anti-diabetic, anti-inflammatory, cardioprotective, hepatoprotective, as well as cholesterol-lowering activities (Zhu 2016; Lv et al. 2017; Ge and Wang 2020). Therefore, Tartary buckwheat tea and alcohol are deeply loved by consumers for health care functions. Overall, buckwheat is a kind of popular food due to its nutrient-rich healthy and low calories, and it is a mode of application in both medicine and food (Huda et al. 2021).

Seeds play a vital role in the whole life cycle of a plant. For annual plants, seeds are the end of the life of female parent, and the start of the next generation. Their stored nutrition does not only provide food for humans and other animals, but also provide energy required for seed germination. Carbohydrates, proteins, and oils are reported as the main three storage in seeds (Weber et al. 2005). Tartary buckwheat accumulates starch ranging from 35.01 to 86.83% (Gao et al. 2016), protein (10 ~ 12%) (Bonafaccia et al. 2003; Guo and Yao 2006), and the flavonoid content is more than 2% (Qin et al. 2010). The process of these nutrition accumulations during seed development is very complex, necessitating studies to reveal the regulation mechanism. Proteome studies during seed filling have been performed in many crops, including rice (Xu et al. 2008), wheat (Cui et al. 2017), maize (wang et al. 2018), mungbean (Kazłowski et al. 2013), lotus (Wang et al. 2016), soybean (Agrawal et al. 2008), among others. Transcriptome analyses in Tartary buckwheat seed development have been conducted (Huang et al. 2017; Li et al. 2019b). However, proteome analyses in Tartary buckwheat seed remain elusive.

In the present study, we carried out a gel-based proteomic analysis of rice Tartary buckwheat seeds at three different development stages, and the dynamic expression of proteins during seed development was uncovered. This study will not only help in expanding the understanding of the regulation mechanism of seed development, but also breed high quality variety of Tartary buckwheat.

## Materials and methods

### Plant materials

A new rice Tartary buckwheat line with thin seed shell, high yield, named “Mi 11”, is derived from the cross progenies of “Jinqiaomai 2” (thick shelled) and “Xiaomiqiao” (thin shelled). These plant materials were cultured in our testing field in autumn under normal field management during the growth periods. Five developmental stages of seeds were taken from emergence to maturity after pollination. Then seed coats were stripped and fixed in formalin-aceto-alcohol (FAA) solution (70% ethanol: glacial acetic acid: 5% formaldehyde = 90: 5: 5) immediately at room temperature for 48 h for further paraffin-embedded sections. Based on the morphology of the seeds and their longitudinal sections as well as observation of the accumulation of starch and proteins, samples for proteomic analysis were collected at three stages, namely pre-filling stage (PS, stage 1 and 2), filling stage (FS, stage 3 and 4), and mature stage (MS, stage 5 and 6) according to a previous study (Huang et al. 2017). Seed coats were stripped on ice, the remaining seeds were frozen with liquid N_2_ and stored at − 80 °C for protein and RNA extraction.

### Paraffin-embedded section and dyeing

After dehydration and paraffin infiltration, samples were embedded in melted paraffin and then frozen at − 20 °C until the paraffin solidified completely. The paraffin blocks were cut into 4-µm-thick longitudinal sections using a microtome. Then, the paraffin section were stained in periodic acid, Schiff’s reagent, and naphthol yellow S according to the instruction of PAS + NS staining kit (Servicebio, Wuhan, China), in order to view the accumulation profile of starch and proteins of “Mi 11” seeds at different developmental stages. After dehydration, the paraffin sections were mounted with resin for observation.

### Total flavanoid measurement

The measurement method of total flavanoid of Tartary buckwheat seeds was done according to that described by Song et al. (2016). Briefly, about 0.1 g frozen sample powder was mixed with 15 mL 70% methanol, then shook at 60 ^o^C, 200 rpm for 2 h. After centrifugation, the extract was diluted to 25 mL with 70% methanol and 1 mL of the diluted mixture was mixed with 2 mL AlCl_3_ (0.1 M) and 3 mL K_2_CH_3_CO_2_ (1 M), filled up to 10 mL with 4 mL 70% methanol. After mixing well and reacting for 30 min, the absorbance of the mixture was measured at 420 nm. Total flavonoid content was calculated using a calibration curve, which was constructed by rutin standard.

### Protein extraction and 2-DE

Total proteins of rice Tartary buckwheat seeds were extracted following the phenol method described by Deng et al. (2015). The obtained protein pellet was vacuum dried and dissolved in a moderate amount of lysis buffer which contained 7 M urea, 2 M thiourea, 4% (w/v) 3-[(3- cholamide propyl) dimethylammonium]-1-propylsulfonate (CHAPS), 65 mM dithiothreitol (DTT), and 0.2% Bio-Lyte pH 3–10, and the soluble protein samples were quantified by Bradford method (Bradford 1976). Each sample was performed with three biological repeats.

Each sample with 0.6 g total protein was adjusted to 350 μL by adding lysis buffer, then subjected to rehydration for 12 h at room temperature by loading onto IPG strips (pH 5–8, linear, Bio-Rad). 2-DE was performed as described by Deng et al. (2015). After running the 2-DE, gels were stainedwith Coomassie brilliant blue (CBB) R-250, then scanned using an Epson Perfection V700 Photo scanner (Epson, China Co., Ltd.), and the resolution of photos was set at 600 dpi. The abundance of protein spots in the gel images was analyzed by PDQuest™ 2-DE software (Bio-Rad).

### In-gel digestion and MALDI-TOF/TOF MS analysis

Protein spots with abundance ≥ twofold changes were excised from 2-DE gels, then washed, distained, dehydrated as described previously (Li et al. 2012), and digested with trypsin as described by Yang et al. (2007). After purification by C_18_ NuTip (Sigma, USA), the concentrated peptide solution was analyzed by Autoflexspeed™ MALDI-TOF-TOF MS (BrukerDalton, Germany) in an MS–MS mode. The parameters were set as follows: the wavelength of UV was 355 nm, a 0.2-kHz laser with an optimum quality resolution of 1500 Da was applied, and the range of scan was 700–3200 Da. All the mass spectrogram of samples were obtained in default mode. The flexAnalysis software (BrukerDalton) was used to process MS/MS spectra. Then, the MS–MS data were searched against the database of Tartary buckwheat proteins which was built using the predicted proteins by Tartary buckwheat genome sequences (ftp://ftp.kazusa.or.jp/pub/buckwheat/). The parameters of search criteria were set as follows: peptide mass range, 800–4000 Da; MS tolerance, 100 ppm, and MS–MS tolerance, 0.6 Da; Peptide charge was set at + 1; Globalmodification and variable modification were set as carbamidomethy and Oxsidation, respectively. Proteins that reached significance > 60 with *P* < 0.05 were regarded as significant positive identification, and the matches with the highest scores were chosen as the final results.

### Cluster analysis, functional categories, and metabolic pathway analyses

Cluster analysis was performed by Genesis software using the method of hierarchical cluster analysis and complete linkage.

Functional categories of the identified DEP were assigned using MAPMAN (http://mapman.mpimpgolm.mpg.de/general/ora/ora.ht-ml) described by Deng et al. (2015). Metabolic pathway analyses were analyzed by the KEGG GENES database (http://www.genome.jp/kegg/genes.html).

### RNA extraction and qRT-PCR analysis

Total RNA of Tartary buckwheat seeds was extracted with GREENspin Plus Plant RNA kit (ZoManBio, Beijing, China) according to the manufacturer’s guidelines. Reverse transcription was performed using a First Strand cDNA Synthesis Kit (TOYOBO, Beijing, China). The expression profiles of some structure genes in the flavonoid pathway were analyzed by qRT-PCR as described previously (Deng et al. 2021), each sample identical with the sample for the proteome assay was performed in three biological replicates. The Tartary buckwheat *actin* gene (*FtPinG0005405200.01*) (Li et al. 2019b) was used as the reference, and the relative expression level of selected genes was calculated via the 2^−ΔΔCt^ method. The specific primers of these genes are listed in Table S1.

## Results and discussion

### Morphological changes during Tartary buckwheat seed development

**Fig. 1:**
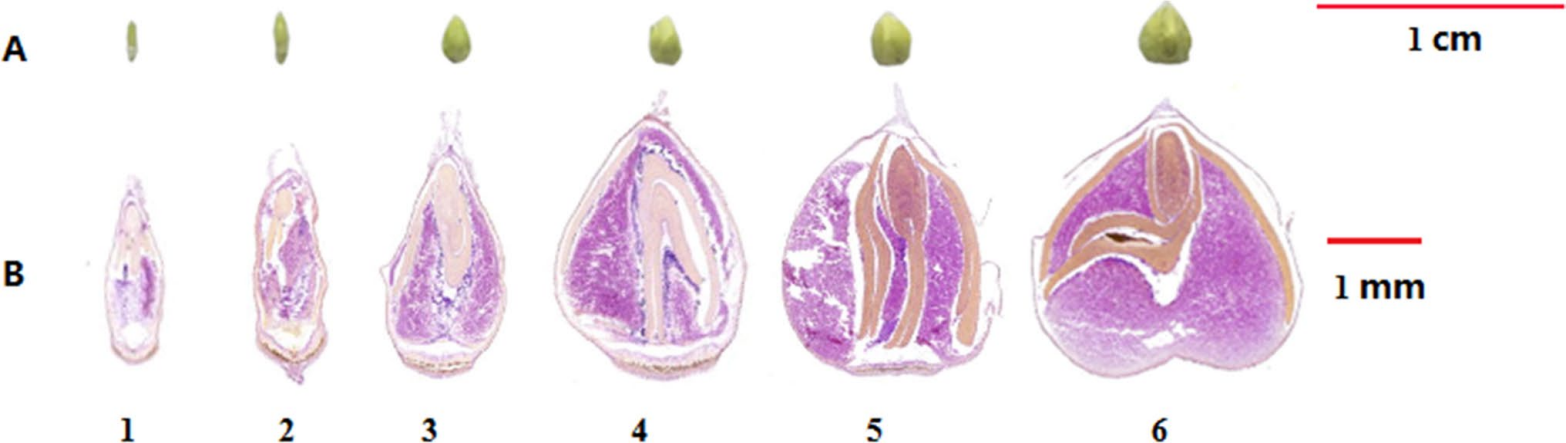
The morphology of six developmental stages of ‘’Mi 11 seed (**A**) and their longitudinal section (brown represents dyed for protein, and purple means dyed for starch) (**B**)

Six different developmental stages of ‘Mi 11’ seeds from young fruit to maturity were collected, and seed coats were shelled (Fig. 1A). To further reveal different reserves accumulation, light microscope observations were used on the longitudinal section of these samples. At the first two stages, the seed surfaces were symmetrical, but the embryo developed gradually from the top of the seed to the other side. As the two main nutritional compositions in buckwheat seed, starch started to emerge in the endosperm, while proteins were mainly accumulated in the embryo (Fig. 1B). At the third and fourth stage, the surface of the seed started to drum up and became fragile, while the embryo continued to develop and accumulated more proteins and starch massively accumulated. From stage 5 to stage 6, the seed expanded filling the whole seed shell and became strong.

At these two stages, not only the seed size obviously changed, but also protein mainly accumulated with the development of the embryo, and starch gradually filled the whole endosperm. Based on the changes of morphology and deposition of storage reserves of ‘Mi 11’ seeds, samples could be classified into three phases, including pre-filling stage (PS, stage 1 and 2), filling stage (FS, stage 3 and 4), and mature stage (MS, stage 5 and 6) which is consistent to the previous report (Huang et al. 2017).

### Comparative proteomic analysis of Tartary buckwheat seeds at various stages of development

**Fig. 2:**
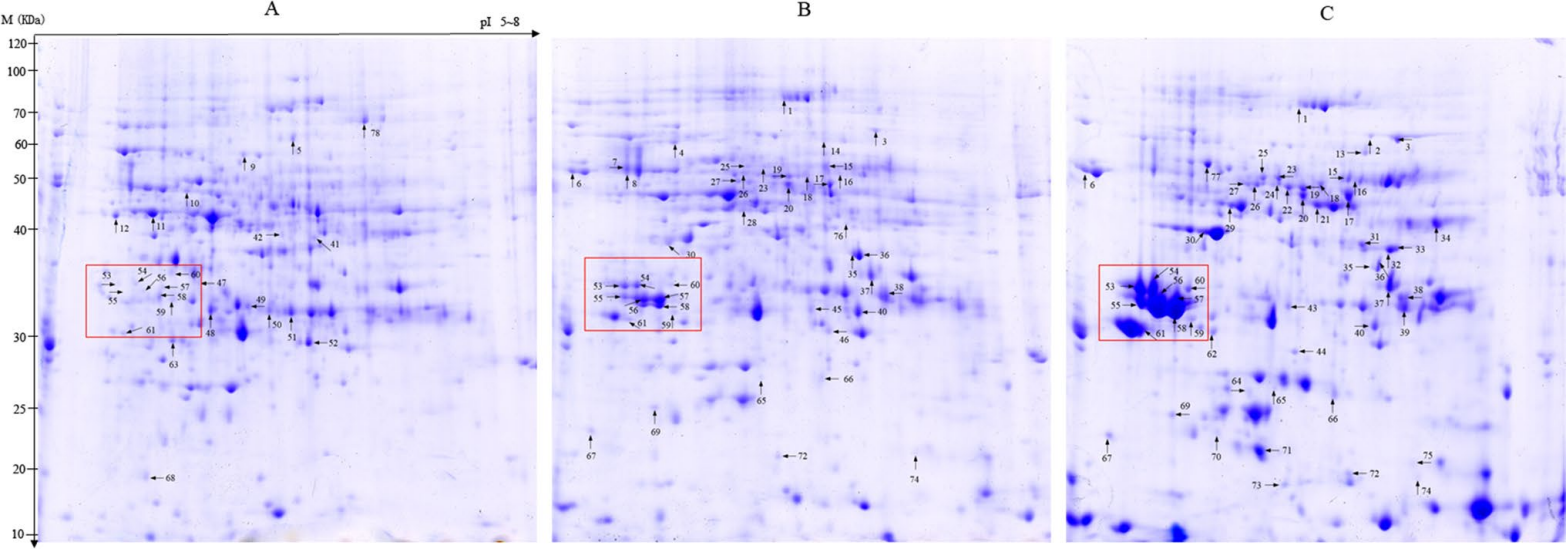
Representative 2-DE gel images of ‘Mi 11’ seed proteins from three different stages. (**A**) Pre-filling stage (PS), (**B**) filling stage (FS), and (**C**) mature stage (MS). Arrows and numbers stand for the differentially expressed protein spots and their ID

To uncover the mechanism of dynamic changes during Tartary buckwheat seed development, comparative proteomic analysis was monitored on the developing “Mi 11” seeds at three stages including pre-filling stage (PS), filling stage (FS), and mature stage (MS) by 2-DE in the *pI* range 5–8. Approximately 800 protein spots were detected reproducibly in all three biological replicates gels stained by CBB R-250. Most of them were distributed at the molecular weight range of 15–100 KDa (Fig. 2). Several protein spots showed significant differential expressions with the development stage (highlighted in the red box of Fig. 2). Quantitative analysis of protein spots from three biological replicates of each sample was conducted by PDQuest™ software. Totally, 78 protein spots showing a more than two-fold change in expression level in at least one developmental stage of the seeds were confirmed as differentially expressed proteins (DEPs) (Fig. 2), and that they may play roles in seed development of Tartary buckwheat. Some proteins with inconsonant pIs or molecular weights or both, but were identified as the same protein. For example, five spots including 19, 22, 55, 57, and 58 were all identified as 11 S seed storage protein (FtPinG0000917200.01) (Table S2). This might be due to alternative splicing, allelic variation, post-translation modification, and so on (Dafny-Yelin et al. 2005; Deng et al. 2015). Additionally, spot 54 (FtPinG0000291200.01), spot 56 (FtPinG0000917700.01), spot 61(FtPinG0009373700.01) etc. were also identified as 11 S seed storage protein, this was because they are all members belonging to the protein family of 11 S seed storage proteins (Deng et al. 2017).

**Fig. 3:**
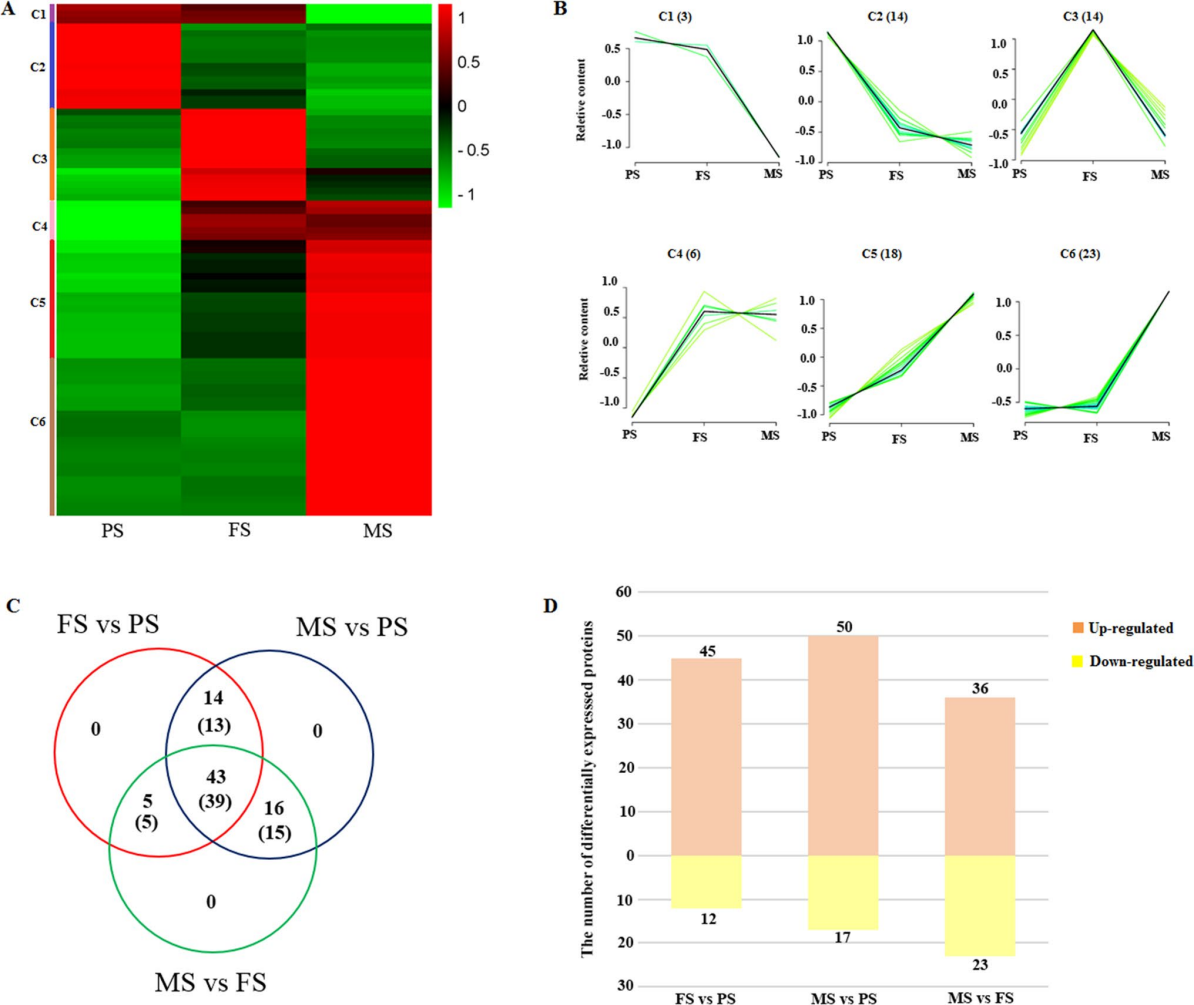
Differentially expressed proteins profile analysis. (**A**) Clustering heat map of all differentially expressed proteins, and (**B**) sequence charts of differentially expressed proteins in developing buckwheat seeds for each cluster depicted in A. (**C**) Venn diagram of three comparison groups (the figures outside the brackets are the number of differentially expressed proteins, and those in the brackets are the number of identified DEPs by MALDI-TOF/TOF MS). (**D**) The number of upregulated and down-regulated identified DEPs of three comparison groups

These DEPs were divided into six clusters based on their expression profiles (Fig. 3A, B). Cluster 1 (C1) only contained 3 proteins which were all slightly declining in the seeds filling stage (FS), and decreased sharply at the maturity stage (MS) seeds compared with pre-filling stage (FS) seeds. DEPs in C2 (14) were significantly downregulated in FS seeds which then decreased slowly at MS seeds. C3 also contained 14 DEPs, which were upregulated from PS to FS seeds, then downregulated at MS seeds. C4 had 6 proteins, most of them being upregulated at FS seeds, then showed no significant change at MS seeds. C5 contained the second majority of DEPs (18), which were gently elevated from PS to FS seeds, and upregulated faster at MS seeds. C6 contained the greatest number of DEPs (23), most of them exhibited no significant change between FS and PS seeds, but rapidly increased at MS seeds (Fig. 3A, B).

These DEPs were identified by matrix-assisted laser desorption/ionization tandem time of flight mass spectrometry (MALDI-TOF/TOF MS). Seventy-two protein spots were successfully identified with about 95% identification rate, and 6 protein spots (10, 20, 44, 67, 69, and 75) were not identified with no hits found (Table S2). Three comparison groups, FS vs. PS, MS vs. PS, and MS vs. FS, shared 39 identified DEPs (Fig. 3C), indicating that most of the DEPs play roles throughout the whole development of Tartary buckwheat seed. Each DEP showed a differential expression level in at least two comparison groups (Fig. 3C). Among these identified DEPs, 57, 67, and 59 members in FS vs. PS, MS vs. PS, and MS vs. FS comparison group, respectively, and upregulated DEPs in each group all showed more abundant than those down-regulated (Fig. 3D), which suggested that most of the proteins, especially enzymes improved their activity with Tartary buckwheat seed developing progress to promote the formation of seeds.

### Functional categories of identified DEPs

**Fig. 4:**
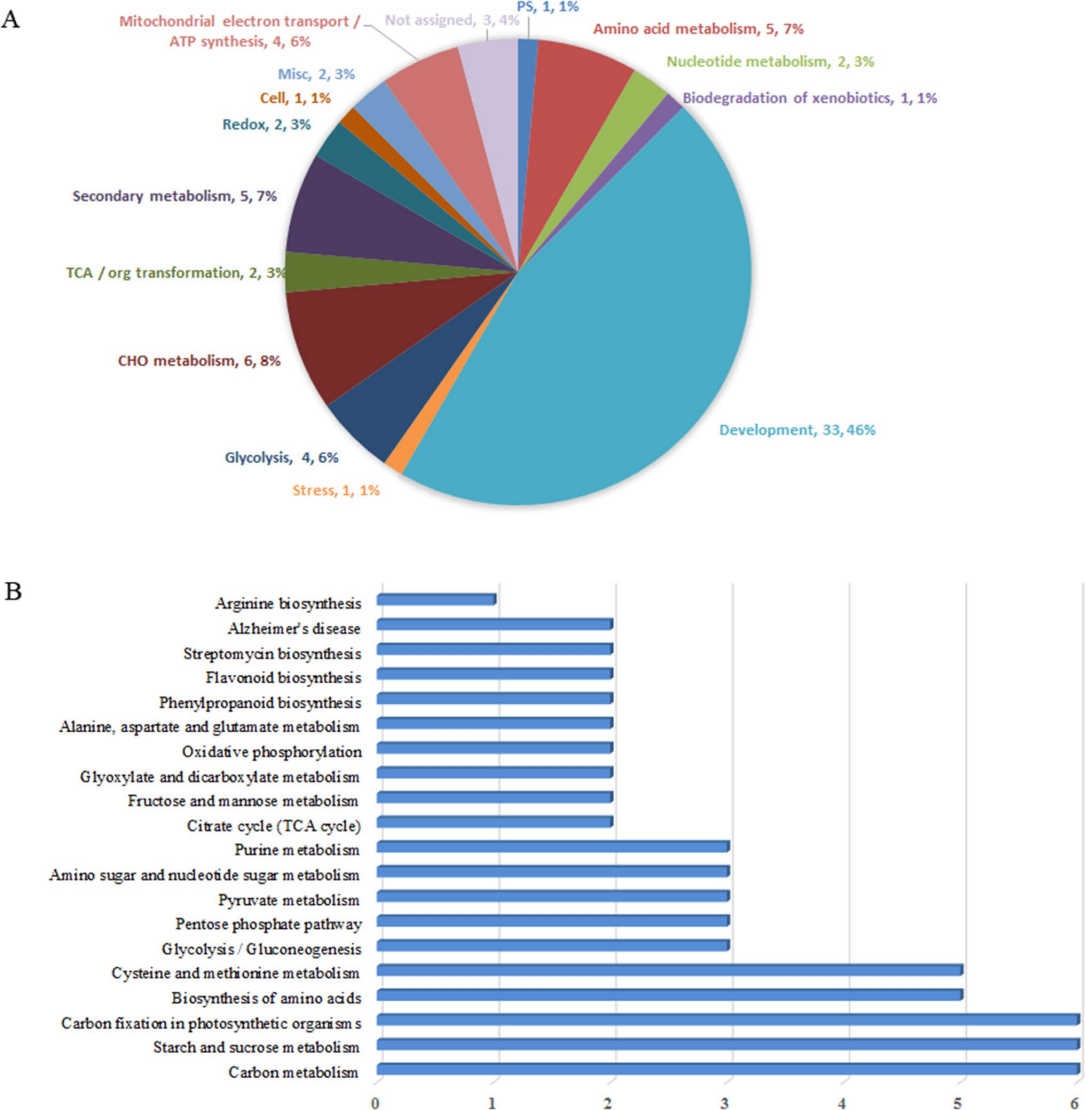
GO and KEGG analysis of DEPs. (**A**) Functional categorization and their number as well as ratios of these identified proteins based on GO analysis. (**B**) The top 20 pathways by KEGG pathway analysis

These identified DEPs were classified into 15 functional groups according to the gene ontology (GO) annotation (Fig. 4A, Table S2). Among them, proteins that were involved in development were the majority (33 DEPs, occupying 46% of total DEPs), which mainly contained seed storage protein, including 11 S seed storage protein and vicilin. The numbers of other protein functional groups were all less than ten, six proteins were involved in CHO metabolism, five proteins were involved in secondary metabolism and amino acid metabolism, respectively, and four proteins were related to glycolysis and mitochondrial electron transport/ ATP synthesis, respectively. Only a small number of proteins were involved in TCA/ org transformation, redox, cell, misc, ps, biodegradation of xenobiotics, and nucleotide metabolism. Three DEPs were not assigned (Fig. 4A, Table S2).

When these DEPs were subjected to KEGG analysis, the top 20 pathways were chosen (Fig. 4B). Among them, proteins enriched in carbon metabolism, starch, and sucrose metabolism, and carbon fixation in photosynthetic organisms contained the most abundant enzymes identified in this study, followed by biosynthesis of amino acids, cysteine, and methionine metabolism pathways. Meanwhile, proteins involved in the metabolism of plant’s basic substances including glycolysis/gluconeogenesis, pentose phosphate pathway, pyruvate metabolism, amino sugar, and nucleotide sugar metabolism, and purine metabolism only contained 3 members for each pathway. Other pathways only contained no more than 2 proteins, including several primary metabolites pathways, such as amino acid metabolism, TCA cycle, glyoxylate, and dicarboxylate metabolism. In addition, some proteins were enriched in secondary metabolism pathways, for example, phenylpropanoid biosynthesis and flavonoid biosynthesis pathways (Fig. 4B).

### Proteins involved in starch and seed storage protein accumulation

Starch is one important component of buckwheat seed, which accumulates in endosperm with seed development (Fig. 1B). Starch biosynthesis pathway has been well researched in some cereals, including rice (Baysal et al. 2020; Dong et al. 2019b), maize (Dong et al. 2019a, b), wheat (Gao et al. 2021; Guo et al. 2020), and barley (Zhong et al. 2021). It starts from sucrose, which is catalyzed by sucrose synthase (SUS), UDP glucose pyrophosphorylase (UGPase), UDP glucose pyrophosphorylase (AGPase), and granule bound starch synthase (GBSS) systematically to produce amylose, which can be converted to amylopectin through catalytic reaction by starch synthase (SS), starch-branching enzyme (SBE) and debranching enzyme (DEB). Here, we identified a granule bound starch synthase GBSS1, (FtPinG0000359400.01), which was reported to be a crucial enzyme that directly affects amylose content and total starch quality (Nougué et al. 2014), and has been identified in Tartary buckwheat (Wang et al. 2014). Previous studies have identified several *GBSS1* genes, which expressed differentially in developing Tartary buckwheat and common buckwheat seeds (Huang et al. 2017; Li et al. 2019a). However, FtPinG0000359400.01 exhibited a continuously increasing trend from PS to MS seed, especially, sharply increasing from FS to MS, which was consistent with starch accumulation. Therefore, GBSS1 was considered the speed-limiting enzyme for the starch biosynthesis pathway of Tartary buckwheat seed.

Seed storage proteins are the second largest nutrient component of buckwheat, accounting for 9.22–14.55% of dry seed weight (Raina and Gupta 2015), which is higher than that of rice (He et al. 2021). Like other crops, buckwheat seed storage proteins are also divided into four categories, water-soluble albumin, alcohol-soluble prolamine, salt-soluble globulin, and acidic or basic solution-soluble glutelin. We identified two kinds of differentially expressed globulins during buckwheat seed filling, 11 S and vicilin (also named 7S globulin), both of them having 8 and 2 members, respectively, and most of them were upregulated with seed development process (Table S2). Among them, several 11 S seed storage proteins remarkably accumulated during seed development, which could be detected in gel images from PS to MS (protein spots in red box) (Fig. 2). 13 S and 7 S globulin proteins were identified differentially expressed in transcriptome analysis (Huang et al. 2017), but the expression profile of 7 S globulin in the transcriptional level was on the contrary with that in the protein expression level identified here. Although the previous study suggested that albumin was the predominant protein fraction in Tartary buckwheat seeds (Guo and Yao 2006), globulins expressed significantly differentially during seed development and seemed to be the dominant protein composition in our study. Therefore, globulin may be the predominant protein component in “Mi 11” Tartary buckwheat cultivar and play a vital role in seed development as well as formation.

### Proteins involved in the flavonoid biosynthesis pathway

**Fig. 5:**
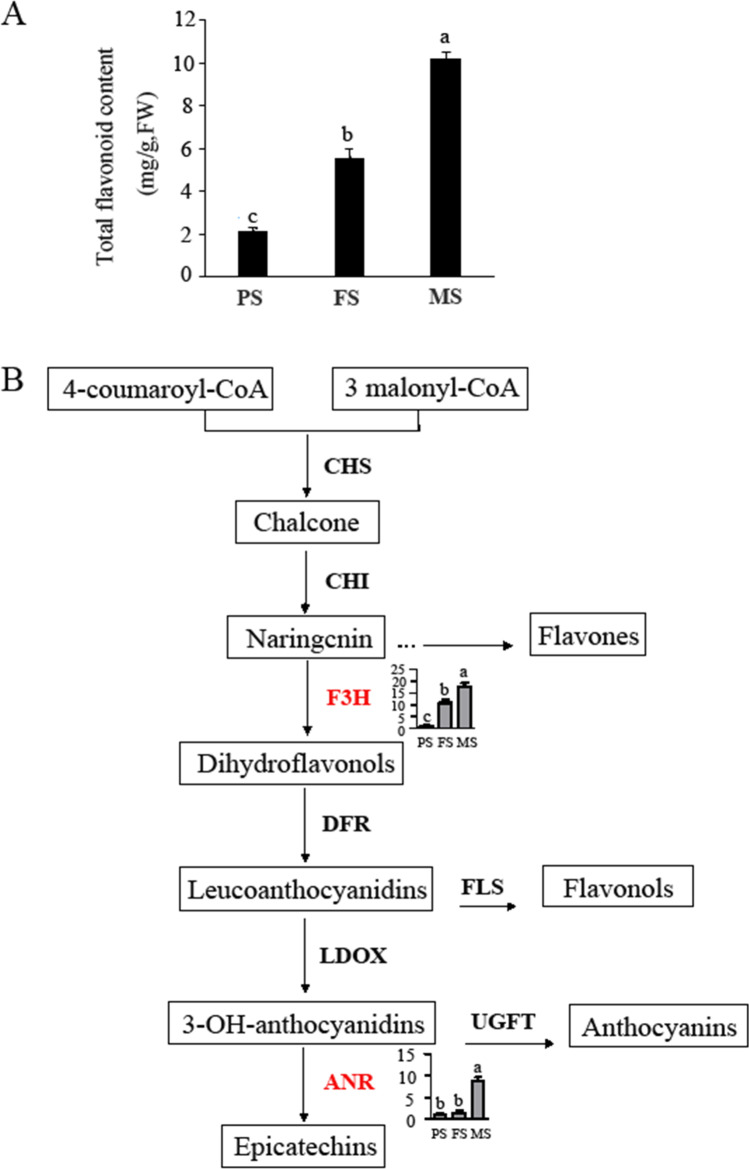
Total flavonoid content and DEPs involved in flavonoids biosynthesis pathway. (**A**) Total flavonoid content of seeds in three different developmental stages. FW, fresh weight. The values represent means ± SD (*p* < 0.05). (**B**) KEGG flavonoids biosynthesis pathway and the relative abundance of two enzymes. CHS, chalcone synthase; CHI, chalcone isomerase; F3H, flavanone 3-hydroxylase; DFR, dihydroflavonol 4-reductase; UFGT, flavonoid 3-O-glucosyltransferase; FLS, flavonol synthase; ANR, anthocyanidin reductase. PS, pre-filling stage seeds; FS, filling stage seeds and MS, mature stage seeds

**Fig. 6:**
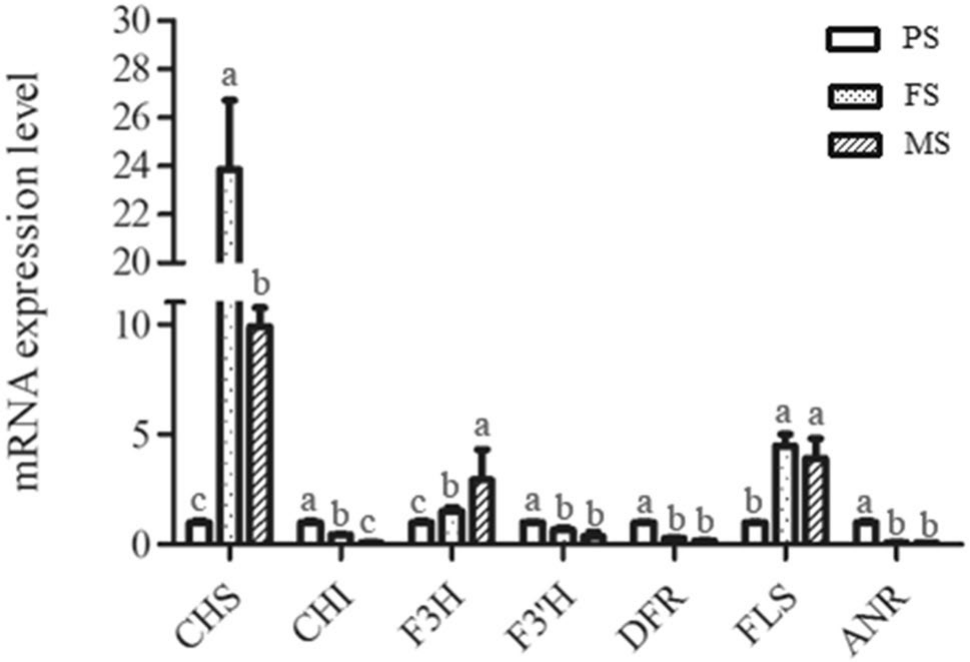
Relative expression level of the genes in the flavonoid biosynthetic pathway in developing Tartary buckwheat seeds. The values represent the means ± SD (*p* < 0.05)

Flavonoids are the main bioactive component of Tartary buckwheat that other common crops lack. Due to these high flavonoids level, Tartary buckwheat possesses diverse health and pharmaceutical properties, and is considered as one potentially valuable food source, and it has therefore drawn more and more attention (Sinkovič et al. 2020; Huda et al. 2021). Genes in flavonoid biosynthesis pathway in Tartary buckwheat seed have been researched on transcriptional levels, and many structural genes were indicated differentially expressed during seed development (Huang et al. 2017; Li et al. 2019b). Here, the total flavonoid content of three developmental stages of seeds was detected, which showed a sustained rising trend from PS to MS seeds and reached the highest in MS seeds (Fig. 5A). Meanwhile, two enzymes, one flavanone 3-hydroxylase (F3H, FtPinG0008251700.01) and one anthocyanidin reductase (ANR, FtPinG0007896600.01), which were enriched in the flavonoid biosynthesis pathway, were identified (Table S2, Fig. 5B). F3H continued to increase from PS to MS seeds, its expression pattern was consistent with flavonoids accumulation. However, ANR showed no significant change from PS to FS and sharply increased in MS seeds, which was incongruent with flavonoids accumulation. Meanwhile, the expression of genes in the flavonoid pathway at the mRNA level was analyzed by qRT-PCR. As shown in Fig. 6, *CHS* and *FLS* expressed highest in FS seeds, followed by MS seeds, and had lowest expression level in PS seeds. While four genes *CHI*, *F3’H*, *DFR* and *ANR* had the highest expression level in PS seeds, then nearly all of them sustained a decrease in FS and MS seeds with most of them showing no significant difference between FS and MS seeds, which were opposite with flavonoid accumulation pattern. However, only the expression profile of *F3H* was consistent with its protein expression level as well as flavonoid accumulation. Taken together, F3H was speculated to be the key enzyme in the flavonoid biosynthesis pathway of “Mi 11” Tartary buckwheat seeds.

Our results were similar with the transcriptome results reported in Tartary buckwheat sprouts previously (Zhang et al. 2019), which indicated that *F3’H* gene, encoding another flavanone hydroxylase and being important in the production of rich flavonol components, was the key gene in rutin accumulation. Notably, this gene is activated by an MYB116 transcriptional factor (Zhang et al. 2019).

## Conclusion

The development of crop seeds is divided into three stages, pre-filling stage, filling stage, and mature stage. According to microscopic observations, very clear dynamic changes of the outer shape of developing seeds, inner embryo, and endosperm as well as the accumulation of starch and protein could be observed. Proteomic analysis of these three developmental stages of Tartary buckwheat seeds was performed, which revealed that GBSS1 might be the key enzyme that controls starch accumulation, and that 11S seed storage protein and vicilin were the main globulin and the predominant protein component of tartar buckwheat protein. Additionally, F3H might be the speed-limiting enzyme in the flavonoid biosynthesis pathway in Tartary buckwheat seed. Our results uncovered the regulatory mechanism of the main nutritional accumulation of buckwheat seed during differential development stages and will help in elucidating other aspects of the developmental biology of Tartary buckwheat.

## Supplementary Information

Below is the link to the electronic supplementary material.

Supplementary file1 (DOCX 15 KB) Table S1 Primers for qRT-PCR analysis

Supplementary file2 (DOCX 37 KB) Table S2 The information of differentially expressed proteins among different developmental stages of Tartary buckwheat seeds
