# Supplementary material for: Comparative proteomic analyses of Tartary buckwheat (Fagopyrum tataricum) seeds at three stages of development
Source: Funct Integr Genomics. 2022 Nov 11;22(6):1449–58. doi: 10.1007/s10142-022-00912-1 (PMC9701650; doi:10.1007/s10142-022-00912-1)
Supplement: Supplementary file 1 — Supplementary file1 (DOCX 15 KB) Table S1 Primers for qRT-PCR analysis [file 10142_2022_912_MOESM1_ESM.docx]

**Table S1**. Primers for qRT-PCR analysis.

| Gene ID | Forward primer (5’-3’) | Reverse primer (5’-3’) | Product length (bp) |
| --- | --- | --- | --- |
| FtPinG0008131000.01 (*FtCHS*) | GAGAAGATGAAGGCGACGAG | CGAACAAGACTCCCCACTCT | 158 |
| FtPinG0002790600.01 (*FtCHI*) | GAATTGCGTTGCTATTTG | TGGCGATTCTGCTTCTCA | 221 |
| FtPinG0008251700.0 (*FtF3H*) | AGCAAAGCCCTCAACACT | TCCAGCGAGCGAGATTAC | 136 |
| FtPinG0002353900.01 (*FtF3'H*) | ACATCCCAAAGAACGCCACC | TTCCCTCTAACATCCGCACC | 147 |
| FtPinG0006907100.01 (*FtFLS*) | TCCACACTCACCATCCTTGT | CCTTGTACTTTCCGTTGCTC | 145 |
| FtPinG0002371500.01 (*FtDFR*) | TAAGATGACTGGCTGGATG | GAGTTGGGATAATGCTAATG | 107 |
| FtPinG0006606900.01 (*FtUGT*) | GAGTTGAGGTCGAAGGAGGT | GTTCCGAGCAGAGCTTCCAT | 142 |
| FtPinG0007896600.01 (*FtANR*) | TTTGTAGCGGAGAAGGAGTC | TGAACCCCTCTTTGGTAAGC' | 187 |
| FtPinG0005405200.01 (*FtActin*) | ATGTTCACTACCACCGCTGA | TGAACCTCTCAGCACCAATC | 172 |
