# Supplementary material for: Comparative proteomic analyses of Tartary buckwheat (Fagopyrum tataricum) seeds at three stages of development
Source: Funct Integr Genomics. 2022 Nov 11;22(6):1449–58. doi: 10.1007/s10142-022-00912-1 (PMC9701650; doi:10.1007/s10142-022-00912-1)
Supplement: Supplementary file 2 — Supplementary file2 (DOCX 37 KB) Table S2 The information of differentially expressed proteins among different developmental stages of Tartary buckwheat seeds [file 10142_2022_912_MOESM2_ESM.docx]

**Table S2** The informations of differentially expressed proteins among different developmental stages of Tartary buckwheat seeds.

| SpotNo.^a^ | Accession | The.^b^ MW/pI（KD） | Exp.^c^ MW/pI （KD） | Score | SC^d^ (%) | Description | Homelogs in TAIR | Id^e^ (%) | FC^f^(FS/PS)  Mean | FC (MS/PS)  Mean |
| --- | --- | --- | --- | --- | --- | --- | --- | --- | --- | --- |
| **PS** | | | | | | | | | | |
| 5 | FtPinG0000269000.01 | 64.9/6.22 | 69.0/6.52 | 252 | 26 | Transketolase, thiamine diphosphate binding domain | AT2G45290 | 81 | 0.15 | 0.09 |
| **Amino acid metabolism** | | | | | | | | | | |
| 36 | FtPinG0005027600.01 | 42.6/6.13 | 39.1/6.89 | 379 | 65 | Amino transferase class I and II | AT5G11520 | 82 | 11.28 | 5.13 |
| 35 | FtPinG0005027600.01 | 37.4/6.13 | 39.1/6.86 | 309 | 57 | Amino transferase class I and II | AT5G11520 | 82 | 14.03 | 3.11 |
| 15 | FtPinG0006411700.01 | 54.8/5.97 | 60.4/6.71 | 243 | 32 | Adenosylhomocystei-nase | AT4G13940 | 46 | 29.08 | 87.69 |
| 16 | FtPinG0006411700.01 | 51.6/6.00 | 57.3/6.80 | 201 | 37 | Adenosylhomocystei-nase | AT4G13940 | 52 | 2.85 | 7.19 |
| 78 | FtPinG0000859500.01 | 68.4/6.09 | 75.0/6.96 | 96 | 13 | Cobalamin-independent methionine synthase | AT5G17920 | 86 | 0.07 | 0.02 |
| **Nucleotide metabolism** | | | | | | | | | | |
| 47 | FtPinG0007297600.01 | 36.8/5.47 | 38.7/5.93 | 366 | 39 | Adenosine kinase | AT5G03300 | 81 | 0.28 | 0.09 |
| 41 | FtPinG0009386400.01 | 49.4/8.31 | 44.3/6.64 | 464 | 43 | Adenylosuccinate synthetase [purA]. | AT3G57610 | 69 | 0.31 | 0.03 |
| **Biodegradation of xenobiotics** | | | | | | | | | | |
| 63 | FtPinG0005365700.01 | 29.3/5.35 | 29.1/5.73 | 270 | 46 | Dihydroxybiphenyl dioxygenase | AT1G11840 | 72 | 0.97 | 0.03 |
| **Development** | | | | | | | | | | |
| 54 | FtPinG0000291200.01 | 35.8/5.66 | 34.1/5.51 | 169 | 26 | 11 S seed storage protein | AT5G44120 | 35 | 8.65 | 18.91 |
| 56 | FtPinG0000917700.01 | 34.9/5.76 | 33.0/5.55 | 362 | 31 | 11 S seed storage protein | AT5G44120 | 39 | 23.60 | 32.09 |
| 55 | FtPinG0000917200.01 | 34.6/5.69 | 33.1/5.44 | 130 | 16 | 11 S seed storage protein | AT1G03880 | 39 | 415.75 | 246.25 |
| 61 | FtPinG0009373700.01 | 30.9/5.36 | 32.4/4.46 | 124 | 20 | 11 S seed storage protein | AT5G44120 | 32 | 39.87 | 238.29 |
| 53 | FtPinG0000292500.01 | 35.1/5.63 | 34.1/5.44 | 201 | 34 | 11 S seed storage protein | AT5G44120 | 34 | 5.41 | 19.56 |
| 60 | FtPinG0004859600.01 | 35.1/5.87 | 34.2/5.74 | 377 | 19 | 11 S seed storage protein | AT5G44120 | 42 | 13.99 | 143.11 |
| 57 | FtPinG0000917200.01 | 34.3/5.69 | 33.0/5.65 | 374 | 37 | 11 S seed storage protein | AT1G03880 | 39 | 206.50 | 788.90 |
| 58 | FtPinG0000917200.01 | 34.1/5.69 | 29.2/5.56 | 416 | 37 | 11 S seed storage protein | AT1G03880 | 39 | 44.97 | 120.13 |
| 30 | FtPinG0004859600.01 | 40.1/5.87 | 44.8/5.71 | 423 | 29 | 11 S seed storage protein | AT5G44120 | 42 | 97.14 | 171.68 |
| 70 | FtPinG0000291200.01 | 22.9/5.66 | 21.3/5.92 | 74 | 14 | 11 S seed storage protein | AT5G44120 | 35 | 1.46 | 9.07 |
| 64 | FtPinG0004859600.01 | 26.3/5.87 | 24.6/6.07 | 271 | 16 | 11 S seed storage protein | AT5G44120 | 42 | 4.66 | 146.03 |
| 29 | FtPinG0009373700.01 | 46.9/5.36 | 56.4/6.03 | 200 | 41 | 11 S seed storage protein | AT5G44120 | 32 | 0.74 | 3.50 |
| 77 | FtPinG0004859600.01 | 55.9/5.87 | 60.6/5.87 | 499 | 32 | 11 S seed storage protein | AT5G44120 | 42 | 24.72 | 79.74 |
| 73 | FtPinG0004859600.01 | 18.9/5.87 | 20.17/6.29 | 153 | 9 | 11 S seed storage protein | AT5G44120 | 42 | 1.02 | 12.91 |
| 24 | FtPinG0000291200.01 | 48.6/5.66 | 59.2/6.25 | 380 | 42 | 11 S seed storage protein | AT5G44120 | 35 | 0.59 | 6.02 |
| 25 | FtPinG0000291200.01 | 53.6/5.66 | 60.0/6.18 | 335 | 48 | 11 S seed storage protein | AT5G44120 | 35 | 8.58 | 68.88 |
| 23 | FtPinG0000291200.01 | 52.8/5.66 | 58.5/6.18 | 515 | 52 | 11 S seed storage protein | AT5G44120 | 35 | 6.03 | 13.54 |
| 22 | FtPinG0000917200.01 | 48.2/5.69 | 55.2/6.34 | 410 | 50 | 11 S seed storage protein | AT1G03880 | 39 | 1.17 | 3.82 |
| 19 | FtPinG0000917200.01 | 59.1/5.69 | 55.2/6.37 | 513 | 57 | 11 S seed storage protein | AT1G03880 | 39 | 7.77 | 52.45 |
| 18 | FtPinG0000917700.01 | 59.1/5.76 | 57.2/6.56 | 247 | 37 | 11 S seed storage protein | AT5G44120 | 39 | 2.39 | 36.64 |
| 59 | FtPinG0001721200.01 | 32.6/5.33 | 31.7/5.73 | 268 | 43 | Seed maturation protein | AT3G22490 | 52 | 6.41 | 47.74 |
| 17 | FtPinG0003652500.01 | 48.5/5.92 | 54.6/6.71 | 152 | 37 | Late embryogenesis abundant protein | AT2G36640 | 34 | 2.20 | 4.59 |
| 71 | FtPinG0002627000.01 | 22.9/6.54 | 21.96/6.17 | 156 | 16 | Vicilin | AT3G58840 | 35 | 1.48 | 1042.31 |
| 72 | FtPinG0002627000.01 | 21.9/6.54 | 21.7/6.67 | 251 | 18 | Vicilin | AT3G58840 | 35 | 12.13 | 44.43 |
| 65 | FtPinG0002626600.01 | 27.6/6.21 | 24.9/6.23 | 166 | 21 | Vicilin | AT2G29940 | 25 | 5.90 | 6.98 |
| 43 | FtPinG0002626600.01 | 32.1/6.21 | 32.7/6.33 | 332 | 22 | Vicilin | AT2G29940 | 25 | 3.30 | 7.90 |
| 21 | FtPinG0002626600.01 | 47.6/6.21 | 51.0/6.48 | 415 | 31 | Vicilin | AT2G29940 | 25 | 13.44 | 38.90 |
| 31 | FtPinG0002626600.01 | 48.6/6.21 | 42.3/5.47 | 244 | 24 | Vicilin | AT2G29940 | 25 | 0.43 | 44.10 |
| 76 | FtPinG0002626600.01 | 40.6/6.21 | 45.1/6.82 | 215 | 24 | Vicilin | AT2G29940 | 25 | 3.33 | 0.35 |
| 74 | FtPinG0002626600.01 | 21.6/6.21 | 20.4/7.23 | 143 | 16 | Vicilin | AT2G29940 | 25 | 3.42 | 3.54 |
| 32 | FtPinG0002626600.01 | 38.6/6.21 | 41.2/6.94 | 347 | 30 | Vicilin | AT2G29940 | 25 | 3.77 | 146.36 |
| 33 | FtPinG0002626600.01 | 39.6/6.21 | 41.2/6.97 | 338 | 28 | Vicilin | AT2G29940 | 25 | 0.99 | 100.75 |
| 34 | FtPinG0002627000.01 | 40.9/6.54 | 43.4/7.24 | 416 | 36 | Vicilin, | AT3G58840 | 35 | 1.61 | 5.37 |
| **Stress** | | | | | | | | | | |
| 9 | FtPinG0009506200.01 | 58.0/4.95 | 65.8/6.22 | 73 | 6 | Tetratricopeptide repeats | AT1G62740 | 58 | 0.34 | 0.27 |
| **Glycolysis** | | | | | | | | | | |
| 13 | FtPinG0005517700.01 | 60.2/5.69 | 63.0/6.82 | 211 | 30 | Alpha-D-phosphohexomutase superfamily | AT1G70730 | 81 | 0.74 | 3.61 |
| 14 | FtPinG0005517700.01 | 60.2/5.69 | 67.0/6.67 | 159 | 33 | Alpha-D-phosphohexomutase superfamily | AT1G70730 | 81 | 22.71 | 7.45 |
| 49 | FtPinG0002549200.01 | 34.7/5.77 | 33.8/6.06 | 426 | 60 | Fructose-bisphosphate aldolase, class-I | AT2G36460 | 85 | 0.31 | 0.29 |
| 48 | FtPinG0000595900.01 | 31.5/6.18 | 29.8/7.02 | 278 | 48 | Glyceraldehyde/Erythr-ose phosphate dehydrogenase family | AT1G13440 | 71 | 0.23 | 0.09 |
| **CHO metabolism** | | | | | | | | | | |
| 28 | FtPinG0001107400.01 | 42.8/6.35 | 49.8/6.15 | 395 | 52 | Nucleotide-diphospho-sugar transferases | AT4G39210 | 72 | 38.15 | 10.14 |
| 26 | FtPinG0000359400.01 | 51.4/8.80 | 56.9/6.18 | 143 | 26 | Granule-bound starch synthase 1 (GBSS1) | AT1G32900 | 62 | 2.99 | 29.56 |
| 45 | FtPinG0007764700.01 | 32.7/8.36 | 32.2/6.58 | 265 | 46 | Ribokinase | AT3G59480 | 62 | 28.71 | 1.08 |
| 1 | FtPinG0000680700.01 | 79.4/3.28 | 82.1/6.47 | 107 | 13 | Zinc finger, CCCH-type | AT3G43190 | 66 | 7.18 | 1.67 |
| 40 | FtPinG0007764700.01 | 32.2/8.36 | 31.6/6.88 | 237 | 46 | Ribokinase | AT3G59480 | 62 | 11.58 | 4.79 |
| 27 | FtPinG0001478700.01 | 49.7/5.41 | 56.4/6.14 | 149 | 23 | Myo-inositol-1-phosphate synthase | AT2G22240 | 90 | 16.82 | 14.57 |
| **Mitochondrial electron transport / ATP synthesis** | | | | | | | | | | |
| 4 | FtPinG0008665900.01 | 60.1/5.20 | 66.4/5.76 | 99 | 9 | P-loop containing nucleoside triphosphate hydrolase | AT1G78900 | 89 | 15.06 | 3.33 |
| 11 | FtPinG0008381400.01 | 42.7/5.47 | 50.6/5.68 | 496 | 60 | P-loop containing nucleoside triphosphate hydrolase | AT5G08680 | 80 | 0.56 | 0.28 |
| 12 | FtPinG0006444800.01 | 42.2/5.12 | 50.7/5.41 | 145 | 31 | 26S proteasome subunit P45/P-loop containing nucleoside triphosphate hydrolase | AT3G05530 | 89 | 0.91 | 0.08 |
| 42 | FtPinG0008000900.01 | 39.3/7.03 | 46.4/6.45 | 131 | 26 | P-loop containing nucleoside triphosphate hydrolase | AT3G19760 | 65 | 0.29 | 0.08 |
| **TCA / org transformation** | | | | | | | | | | |
| 62 | FtPinG0006946200.01 | 31.0/6.26 | 30.2/5.88 | 133 | 15 | Malate dehydrogenase, type 1 | AT3G47520 | 77 | 4.51 | 9.69 |
| 38 | FtPinG0003425400.01 | 34.2/5.59 | 32.2/7.02 | 204 | 59 | Malate dehydrogenase, type 2 | AT1G04410 | 86 | 3.00 | 0.89 |
| **Secondary metabolism** | | | | | | | | | | |
| 39 | FtPinG0007896600.01 | 32.7/5017 | 33.7/5.99 | 224 | 19 | Anthocyanidin reductase | AT1G61720 | 62 | 1.45 | 8.86 |
| 37 | FtPinG0008251700.01 | 33.3/5.55 | 34.25/6.26 | 321 | 58 | Flavanone 3-hydroxylase | AT3G51240 | 85 | 10.92 | 17.45 |
| 51 | FtPinG0003320700.01 | 32.8/5.80 | 33.5/6.48 | 313 | 47 | O-methyltransferase, family 2 | AT1G77520 | 36 | 0.25 | 0.08 |
| 50 | FtPinG0003320700.01 | 32.8/5.80 | 33.5/6.39 | 261 | 32 | O-methyltransferase, family 2 | AT1G77520 | 36 | 0.82 | 0.11 |
| 46 | FtPinG0009234700.01 | 30.7/5.94 | 30.2/6.69 | 359 | 59 | NAD-dependent epimerase/dehydratase N-terminal domain | AT5G58490 | 66 | 3.80 | 0.92 |
| **Redox** | | | | | | | | | | |
| 6 | FtPinG0002162800.01 | 52.5/4.88 | 58.2/5.13 | 283 | 56 | Thioredoxin | AT1G21750 | 69 | 20.22 | 19.67 |
| 66 | FtPinG0001375100.01 | 26.9/6.09 | 25.9/6.92 | 205 | 62 | Glutathione S-transferase, C-terminal-like | AT1G75270 | 71 | 0.99 | 59.59 |
| **Cell** | | | | | | | | | | |
| 52 | FtPinG0009693600.01 | 29.3/5.98 | 30.3/6.58 | 200 | 31 | Annexin | AT5G12380 | 41 | 0.23 | 0.30 |
| **Misc** | | | | | | | | | | |
| 7 | FtPinG0004331000.01 | 52.3/5.19 | 58.8/5.52 | 124 | 22 | Glycoside hydrolase, family 1 | AT2G44480 | 51 | 22.60 | 0.56 |
| 8 | FtPinG0004331000.01 | 52.1/5.19 | 57.6/5.52 | 117 | 27 | Glycoside hydrolase, family 1 | AT2G44480 | 51 | 13.37 | 0.47 |
| **Not assigned** | | | | | | | | | | |
| 68 | FtPinG0007316100.01 | 19.2/5.20 | 20.93/5.60 | 258 | 52 | Protein of unknown function, DUF538 | AT4G24130 | 60 | 0.21 | 0.15 |
| 2 | FtPinG0003302500.01 | 61.8/7.67 | 70.2/6.83 | 123 | 21 | Coil | AT2G42560 | 52 | 9.4 | 100.75 |
| 3 | FtPinG0003302500.01 | 62.5/7.67 | 68.6/6.99 | 269 | 35 | Coil | AT2G42560 | 52 | 9.25 | 88.06 |
| **No hits found** | | | | | | | | | | |
| 10 |  | 48.4/5.37 |  |  |  | No hits found |  |  | 0.34 | 0.07 |
| 20 |  | 48.5/5.68 |  |  |  | No hits found |  |  | 6.14 | 5.54 |
| 44 |  | 29.1/6.13 |  |  |  | No hits found |  |  | 5.85 | 30.74 |
| 67 |  | 23.5/4.90 |  |  |  | No hits found |  |  | 72.4 | 161.8 |
| 69 |  | 25.0/5.34 |  |  |  | No hits found |  |  | 105.8 | 213.2 |
| 75 |  | 18.3/6.14 |  |  |  | No hits found |  |  | 0.74 | 3.40 |

Note: a, Spot number in 2-DE gels as shown in Figure 2. b, Theoretical molecular weight and pI. c, Experimental molecular weight and pI. d, Sequence coverage. e, Identity. f, Fold change.
